# Methyl 12-bromo­dehydro­abietate

**DOI:** 10.1107/S1600536810012201

**Published:** 2010-04-28

**Authors:** Hong Gao, Zhan-Qian Song, Shi-Bin Shang

**Affiliations:** a Institute of Chemical Industry of Forest Products, Chinese Academy of Forestry, Nanjing 210042, People’s Republic of China

## Abstract

The title compound [systematic name: (1*R*)-methyl 6-bromo-7-isopropyl-1,4a-dimethyl-1,2,3,4,4a,9,10,10a-octa­hydro­phen­anthrene-1-carboxyl­ate], C_21_H_29_BrO_2_, was synthesized from *N*-bromo­succinimide and methyl dehydro­abietate, which was prepared through an esterification reaction using dehydro­abietic acid and methanol as raw materials. The three six-membered rings adopt planar (mean deviation = 0.002 Å) half-chair and chair conformations. The two cyclo­hexane rings form a *trans* ring junction with the two methyl groups in axial positions. The crystal structure is stabilized by weak inter­molecular C—H⋯O contacts along the *b* axis.

## Related literature

For the isolation of dehydro­abietic acid, see: Halbrook & Lawrence (1966 ▶). For the preparation and use of dehydro­abietic acid derivatives, see: Fonseca *et al.* (2001 ▶); Pan *et al.* (2006 ▶). For the synthesis of the title compound, see: Esteves *et al.* (1999 ▶). For related structures, see: Zhang *et al.* (2006 ▶); Rao *et al.* (2009 ▶).
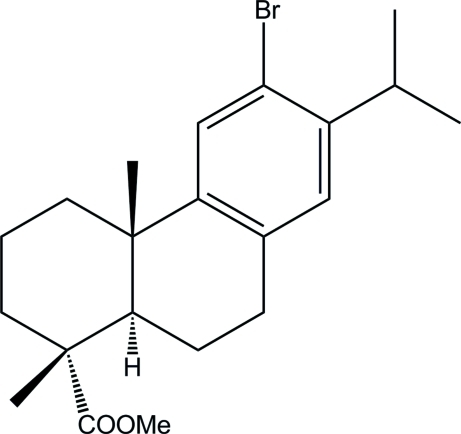


## Experimental

### 

#### Crystal data

C_21_H_29_BrO_2_
*M* *_r_* = 393.35
Monoclinic, 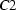

*a* = 13.888 (3) Å
*b* = 6.1260 (12) Å
*c* = 23.382 (5) Å
β = 103.19 (3)°
*V* = 1936.8 (7) Å^3^
*Z* = 4
Mo *K*α radiation
μ = 2.13 mm^−1^
*T* = 293 K
0.30 × 0.20 × 0.20 mm

#### Data collection

Enraf–Nonius CAD-4 diffractometer
Absorption correction: ψ scan (North *et al.*, 1968 ▶) *T*_min_ = 0.567, *T*_max_ = 0.675
3657 measured reflections
3505 independent reflections
2754 reflections with *I* > 2σ(*I*)
*R*_int_ = 0.035
3 standard reflections every 200 reflections  intensity decay: 1%

#### Refinement

*R*[*F* ^2^ > 2σ(*F* ^2^)] = 0.047
*wR*(*F* ^2^) = 0.128
*S* = 1.01
3505 reflections
218 parameters
1 restraint
H-atom parameters constrained
Δρ_max_ = 0.64 e Å^−3^
Δρ_min_ = −0.40 e Å^−3^
Absolute structure: Flack (1983 ▶), 1563 Friedel pairs
Flack parameter: 0.010 (15)

### 

Data collection: *CAD-4 EXPRESS* (Enraf–Nonius, 1994 ▶); cell refinement: *CAD-4 EXPRESS*; data reduction: *XCAD4* (Harms & Wocadlo, 1995 ▶); program(s) used to solve structure: *SHELXS97* (Sheldrick, 2008 ▶); program(s) used to refine structure: *SHELXL97* (Sheldrick, 2008 ▶); molecular graphics: *ORTEP-3* (Farrugia, 1997 ▶); software used to prepare material for publication: *SHELXTL* (Sheldrick, 2008 ▶).

## Supplementary Material

Crystal structure: contains datablocks I, global. DOI: 10.1107/S1600536810012201/zq2035sup1.cif

Structure factors: contains datablocks I. DOI: 10.1107/S1600536810012201/zq2035Isup2.hkl

Additional supplementary materials:  crystallographic information; 3D view; checkCIF report

## Figures and Tables

**Table 1:** Hydrogen-bond geometry (Å, °)

*D*—H⋯*A*	*D*—H	H⋯*A*	*D*⋯*A*	*D*—H⋯*A*
C21—H21*A*⋯O2^i^	0.96	2.72	3.652 (10)	165

Symmetry code: (i) 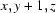.

**Fig. 1.**
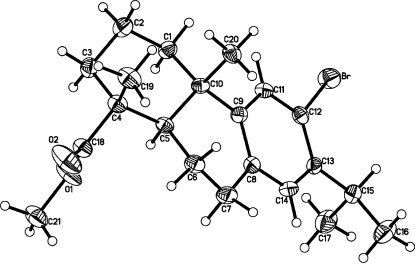
A view of the molecular structure of the title compound, showing displacement ellipsoids at the 30% probability level.

**Fig. 2.**
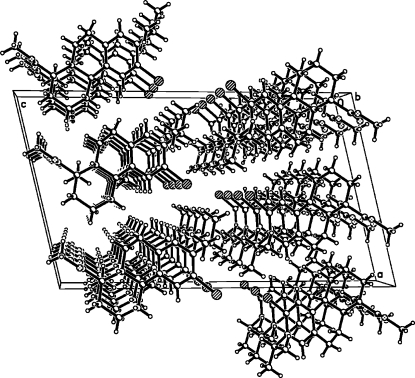
A view of the packing of the title compound along (010).

## supplementary crystallographic information

### Comment

Dehydroabietic acid is a readily obtainable compound, which is isolated from disproportionated rosin by methods of aminaion (Halbrook & Lawrence, 1966). A number of new derivatives of dehydroabietic acid have been prepared (Fonseca *et al.*, 2001). The title compound is one of modified products of dehydroabietic acid, which could be used in synthesis of many fluorescence derivatization reagents (Pan *et al.*, 2006). Although, it has been first prepared by Esteves *et al.*(1999), the crystal structure of the title compound methyl 12-bromo-dehydroabietate was not yet reported. In this work, we present its crystal structure, the molecular structure is shown in Fig. 1. There are three six-membered rings, which adopt planar, half-chair and chair conformations. The two cyclohexane rings form a *trans* ring junction with the two methyl groups in axial positions. The crystal structure is stabilized by weak intermolecular O—H···O contacts and π-π stacking interactions (centroid–centroid distance = 6.126 Å) along the *b* axis (Fig. 2).

### Experimental

*p*-Toluene sulfochloride (4.6 g), potassium carbonate (3.4 g) and methanol anhydrous (20 ml) were mixed and stirred at room temperature for 2 h, then dehydroabietic acid (6.0 g) was added into the mixture to react for 3 h. The mixture was filtered and extracted by ethyl ether and recrystallized by methanol, methyl dehydroabietate (5.3 g) was prepared. Subsequently, methyl dehydroabietate (1.0 g), *N*-bromosuccinimide (1.0 g) and acetonitrile (100 ml) were mixed and stirred for 24 h at room temperature, the precipitate was filtered and recrystallized from methanol. Suitable crystals of the title compound for X-ray diffraction were obtained by slow evaporation of a methanol solution.

### Refinement

All H atoms bonded to the C atoms were placed geometrically at bond distances of 0.93-0.98 Å and included in the refinement in a riding motion approximation with U_iso_(H) = 1.2 or 1.5U_eq_ of the carrier atom.

### Crystal data

**Table:**

C_21_H_29_BrO_2_	*F*(000) = 824
*M**_r_* = 393.35	*D*_x_ = 1.349 Mg m^−^^3^
Monoclinic, *C*2	Melting point: 416 K
Hall symbol: C 2y	Mo *K*α radiation, λ = 0.71073 Å
*a* = 13.888 (3) Å	Cell parameters from 25 reflections
*b* = 6.1260 (12) Å	θ = 10–13°
*c* = 23.382 (5) Å	µ = 2.13 mm^−^^1^
β = 103.19 (3)°	*T* = 293 K
*V* = 1936.8 (7) Å^3^	Rod, colorless
*Z* = 4	0.30 × 0.20 × 0.20 mm

### Data collection

**Table:**

Enraf–Nonius CAD-4 diffractometer	2754 reflections with *I* > 2σ(*I*)
Radiation source: fine-focus sealed tube	*R*_int_ = 0.035
graphite	θ_max_ = 25.3°, θ_min_ = 1.8°
ω/2θ scans	*h* = 0→16
Absorption correction: ψ scan (North *et al.*, 1968)	*k* = −7→7
*T*_min_ = 0.567, *T*_max_ = 0.675	*l* = −28→27
3657 measured reflections	3 standard reflections every 200 reflections
3505 independent reflections	intensity decay: 1%

### Refinement

**Table:**

Refinement on *F*^2^	Hydrogen site location: inferred from neighbouring sites
Least-squares matrix: full	H-atom parameters constrained
*R*[*F*^2^ > 2σ(*F*^2^)] = 0.047	*w* = 1/[σ^2^(*F*_o_^2^) + (0.083*P*)^2^] where *P* = (*F*_o_^2^ + 2*F*_c_^2^)/3
*wR*(*F*^2^) = 0.128	(Δ/σ)_max_ = 0.001
*S* = 1.01	Δρ_max_ = 0.64 e Å^−^^3^
3505 reflections	Δρ_min_ = −0.40 e Å^−^^3^
218 parameters	Extinction correction: *SHELXL97* (Sheldrick, 2008), Fc^*^=kFc[1+0.001xFc^2^λ^3^/sin(2θ)]^-1/4^
1 restraint	Extinction coefficient: 0.0175 (13)
Primary atom site location: structure-invariant direct methods	Absolute structure: Flack (1983), 1563 Friedel pairs
Secondary atom site location: difference Fourier map	Flack parameter: 0.010 (15)

### Special details

**Table:**

Geometry. All esds (except the esd in the dihedral angle between two l.s. planes) are estimated using the full covariance matrix. The cell esds are taken into account individually in the estimation of esds in distances, angles and torsion angles; correlations between esds in cell parameters are only used when they are defined by crystal symmetry. An approximate (isotropic) treatment of cell esds is used for estimating esds involving l.s. planes.
Refinement. Refinement of *F*^2^ against ALL reflections. The weighted *R*-factor wR and goodness of fit *S* are based on *F*^2^, conventional *R*-factors *R* are based on *F*, with *F* set to zero for negative *F*^2^. The threshold expression of *F*^2^ > σ(*F*^2^) is used only for calculating *R*-factors(gt) etc. and is not relevant to the choice of reflections for refinement. *R*-factors based on *F*^2^ are statistically about twice as large as those based on *F*, and *R*- factors based on ALL data will be even larger.

### Fractional atomic coordinates and isotropic or equivalent isotropic displacement parameters (Å^2^)

**Table:**

	*x*	*y*	*z*	*U*_iso_*/*U*_eq_	
Br	0.52734 (4)	0.20204 (10)	0.41747 (2)	0.0569 (3)	
C1	0.4547 (4)	−0.1955 (9)	0.2063 (2)	0.0396 (12)	
H1A	0.4131	−0.2111	0.2342	0.048*	
H1B	0.4601	−0.0409	0.1985	0.048*	
O1	0.6426 (4)	−0.1241 (7)	0.0769 (2)	0.0621 (13)	
O2	0.6696 (4)	−0.4626 (8)	0.0554 (2)	0.0841 (17)	
C2	0.4054 (3)	−0.3087 (14)	0.1497 (2)	0.0478 (12)	
H2A	0.3425	−0.2385	0.1334	0.057*	
H2B	0.3922	−0.4594	0.1581	0.057*	
C3	0.4686 (3)	−0.3032 (13)	0.10451 (19)	0.0448 (11)	
H3A	0.4744	−0.1532	0.0924	0.054*	
H3B	0.4354	−0.3851	0.0701	0.054*	
C4	0.5724 (4)	−0.3975 (9)	0.1272 (2)	0.0387 (12)	
C5	0.6204 (3)	−0.2861 (11)	0.18667 (18)	0.0324 (9)	
H5A	0.6258	−0.1318	0.1768	0.039*	
C6	0.7266 (4)	−0.3588 (9)	0.2118 (2)	0.0423 (14)	
H6A	0.7269	−0.4996	0.2309	0.051*	
H6B	0.7614	−0.3732	0.1805	0.051*	
C7	0.7773 (4)	−0.1913 (11)	0.2557 (3)	0.0502 (15)	
H7A	0.7984	−0.0709	0.2346	0.060*	
H7B	0.8361	−0.2568	0.2801	0.060*	
C8	0.7139 (3)	−0.1013 (9)	0.2953 (2)	0.0361 (11)	
C9	0.6128 (3)	−0.1423 (8)	0.2847 (2)	0.0320 (11)	
C10	0.5593 (3)	−0.2863 (11)	0.23428 (18)	0.0319 (9)	
C11	0.5608 (4)	−0.0505 (9)	0.3232 (2)	0.0357 (11)	
H11A	0.4930	−0.0738	0.3170	0.043*	
C12	0.6087 (4)	0.0746 (8)	0.3703 (2)	0.0339 (11)	
C13	0.7099 (3)	0.1180 (8)	0.3821 (2)	0.0347 (11)	
C14	0.7593 (4)	0.0265 (9)	0.3430 (2)	0.0406 (12)	
H14A	0.8269	0.0517	0.3490	0.049*	
C15	0.7620 (4)	0.2626 (9)	0.4324 (2)	0.0419 (14)	
H15A	0.7239	0.2576	0.4629	0.050*	
C16	0.8676 (4)	0.1866 (15)	0.4603 (3)	0.0641 (15)	
H16A	0.8662	0.0379	0.4730	0.096*	
H16B	0.8952	0.2774	0.4934	0.096*	
H16C	0.9076	0.1969	0.4318	0.096*	
C17	0.7635 (5)	0.5004 (10)	0.4120 (3)	0.0601 (17)	
H17A	0.6973	0.5469	0.3946	0.090*	
H17B	0.8033	0.5113	0.3834	0.090*	
H17C	0.7909	0.5918	0.4450	0.090*	
C18	0.6337 (4)	−0.3401 (9)	0.0833 (2)	0.0398 (14)	
C19	0.5695 (5)	−0.6506 (9)	0.1291 (3)	0.0527 (16)	
H19A	0.5388	−0.7056	0.0908	0.079*	
H19B	0.5321	−0.6966	0.1568	0.079*	
H19C	0.6357	−0.7063	0.1408	0.079*	
C20	0.5494 (4)	−0.5132 (8)	0.2616 (2)	0.0421 (13)	
H20A	0.6140	−0.5693	0.2789	0.063*	
H20B	0.5160	−0.6113	0.2316	0.063*	
H20C	0.5121	−0.4995	0.2913	0.063*	
C21	0.6987 (5)	−0.0491 (12)	0.0371 (3)	0.0620 (18)	
H21A	0.6992	0.1076	0.0368	0.093*	
H21B	0.6697	−0.1020	−0.0016	0.093*	
H21C	0.7653	−0.1021	0.0492	0.093*	

### Atomic displacement parameters (Å^2^)

**Table:**

	*U*^11^	*U*^22^	*U*^33^	*U*^12^	*U*^13^	*U*^23^
Br	0.0488 (3)	0.0726 (4)	0.0544 (3)	−0.0030 (4)	0.0226 (2)	−0.0201 (4)
C1	0.033 (3)	0.045 (3)	0.040 (3)	0.000 (2)	0.008 (2)	−0.005 (2)
O1	0.100 (4)	0.038 (2)	0.066 (3)	−0.015 (2)	0.057 (3)	−0.002 (2)
O2	0.135 (5)	0.052 (3)	0.093 (4)	0.006 (3)	0.082 (4)	−0.007 (3)
C2	0.042 (3)	0.052 (3)	0.048 (3)	−0.001 (4)	0.006 (2)	−0.007 (4)
C3	0.050 (3)	0.048 (3)	0.035 (2)	−0.004 (4)	0.006 (2)	−0.002 (4)
C4	0.046 (3)	0.028 (2)	0.044 (3)	−0.005 (2)	0.015 (2)	−0.005 (2)
C5	0.038 (2)	0.023 (2)	0.038 (2)	−0.003 (3)	0.0137 (18)	0.000 (3)
C6	0.038 (3)	0.044 (4)	0.049 (3)	0.006 (2)	0.018 (2)	−0.001 (2)
C7	0.029 (3)	0.075 (4)	0.052 (3)	0.003 (3)	0.018 (3)	−0.005 (3)
C8	0.033 (3)	0.041 (3)	0.034 (3)	0.000 (2)	0.008 (2)	0.003 (2)
C9	0.028 (3)	0.029 (3)	0.038 (3)	0.004 (2)	0.005 (2)	0.006 (2)
C10	0.030 (2)	0.026 (2)	0.041 (2)	−0.002 (3)	0.0099 (17)	−0.002 (3)
C11	0.030 (3)	0.039 (3)	0.040 (3)	−0.003 (2)	0.013 (2)	0.000 (2)
C12	0.039 (3)	0.031 (3)	0.034 (3)	0.005 (2)	0.013 (2)	0.004 (2)
C13	0.036 (3)	0.034 (3)	0.031 (3)	−0.004 (2)	0.001 (2)	0.006 (2)
C14	0.027 (2)	0.049 (3)	0.045 (3)	−0.006 (2)	0.008 (2)	0.001 (3)
C15	0.043 (3)	0.046 (4)	0.035 (3)	−0.004 (2)	0.005 (2)	−0.001 (2)
C16	0.058 (3)	0.060 (4)	0.063 (3)	−0.006 (4)	−0.008 (3)	−0.006 (4)
C17	0.067 (4)	0.044 (4)	0.062 (4)	−0.008 (3)	−0.002 (3)	−0.003 (3)
C18	0.051 (3)	0.034 (4)	0.039 (3)	−0.004 (3)	0.019 (2)	−0.008 (3)
C19	0.075 (4)	0.028 (3)	0.061 (4)	−0.011 (3)	0.029 (3)	−0.010 (3)
C20	0.053 (3)	0.031 (3)	0.043 (3)	−0.005 (2)	0.013 (3)	0.004 (2)
C21	0.090 (5)	0.052 (4)	0.052 (4)	−0.026 (4)	0.034 (4)	−0.010 (3)

### Geometric parameters (Å, °)

**Table:**

Br—C12	1.916 (5)	C9—C11	1.393 (7)
C1—C2	1.512 (8)	C9—C10	1.524 (7)
C1—C10	1.553 (7)	C10—C20	1.549 (8)
C1—H1A	0.9700	C11—C12	1.382 (7)
C1—H1B	0.9700	C11—H11A	0.9300
O1—C18	1.340 (8)	C12—C13	1.396 (7)
O1—C21	1.420 (7)	C13—C14	1.381 (7)
O2—C18	1.178 (7)	C13—C15	1.517 (7)
C2—C3	1.520 (6)	C14—H14A	0.9300
C2—H2A	0.9700	C15—C17	1.535 (8)
C2—H2B	0.9700	C15—C16	1.535 (8)
C3—C4	1.532 (7)	C15—H15A	0.9800
C3—H3A	0.9700	C16—H16A	0.9600
C3—H3B	0.9700	C16—H16B	0.9600
C4—C18	1.517 (7)	C16—H16C	0.9600
C4—C19	1.552 (8)	C17—H17A	0.9600
C4—C5	1.555 (7)	C17—H17B	0.9600
C5—C6	1.524 (6)	C17—H17C	0.9600
C5—C10	1.547 (5)	C19—H19A	0.9600
C5—H5A	0.9800	C19—H19B	0.9600
C6—C7	1.507 (8)	C19—H19C	0.9600
C6—H6A	0.9700	C20—H20A	0.9600
C6—H6B	0.9700	C20—H20B	0.9600
C7—C8	1.519 (7)	C20—H20C	0.9600
C7—H7A	0.9700	C21—H21A	0.9600
C7—H7B	0.9700	C21—H21B	0.9600
C8—C14	1.391 (7)	C21—H21C	0.9600
C8—C9	1.391 (7)		
			
C2—C1—C10	113.4 (4)	C20—C10—C1	109.5 (4)
C2—C1—H1A	108.9	C5—C10—C1	108.1 (4)
C10—C1—H1A	108.9	C12—C11—C9	120.8 (4)
C2—C1—H1B	108.9	C12—C11—H11A	119.6
C10—C1—H1B	108.9	C9—C11—H11A	119.6
H1A—C1—H1B	107.7	C11—C12—C13	122.9 (4)
C18—O1—C21	118.1 (5)	C11—C12—Br	116.5 (4)
C1—C2—C3	112.3 (5)	C13—C12—Br	120.5 (4)
C1—C2—H2A	109.1	C14—C13—C12	114.9 (5)
C3—C2—H2A	109.1	C14—C13—C15	122.0 (4)
C1—C2—H2B	109.1	C12—C13—C15	123.1 (5)
C3—C2—H2B	109.1	C13—C14—C8	123.8 (4)
H2A—C2—H2B	107.9	C13—C14—H14A	118.1
C2—C3—C4	113.4 (4)	C8—C14—H14A	118.1
C2—C3—H3A	108.9	C13—C15—C17	110.5 (4)
C4—C3—H3A	108.9	C13—C15—C16	113.1 (5)
C2—C3—H3B	108.9	C17—C15—C16	109.9 (5)
C4—C3—H3B	108.9	C13—C15—H15A	107.7
H3A—C3—H3B	107.7	C17—C15—H15A	107.7
C18—C4—C3	107.9 (4)	C16—C15—H15A	107.7
C18—C4—C19	105.9 (4)	C15—C16—H16A	109.5
C3—C4—C19	111.0 (5)	C15—C16—H16B	109.5
C18—C4—C5	108.1 (4)	H16A—C16—H16B	109.5
C3—C4—C5	108.8 (4)	C15—C16—H16C	109.5
C19—C4—C5	115.0 (5)	H16A—C16—H16C	109.5
C6—C5—C10	111.3 (4)	H16B—C16—H16C	109.5
C6—C5—C4	113.4 (4)	C15—C17—H17A	109.5
C10—C5—C4	116.7 (4)	C15—C17—H17B	109.5
C6—C5—H5A	104.7	H17A—C17—H17B	109.5
C10—C5—H5A	104.7	C15—C17—H17C	109.5
C4—C5—H5A	104.7	H17A—C17—H17C	109.5
C7—C6—C5	109.0 (4)	H17B—C17—H17C	109.5
C7—C6—H6A	109.9	O2—C18—O1	120.4 (5)
C5—C6—H6A	109.9	O2—C18—C4	126.9 (5)
C7—C6—H6B	109.9	O1—C18—C4	112.6 (4)
C5—C6—H6B	109.9	C4—C19—H19A	109.5
H6A—C6—H6B	108.3	C4—C19—H19B	109.5
C6—C7—C8	114.6 (4)	H19A—C19—H19B	109.5
C6—C7—H7A	108.6	C4—C19—H19C	109.5
C8—C7—H7A	108.6	H19A—C19—H19C	109.5
C6—C7—H7B	108.6	H19B—C19—H19C	109.5
C8—C7—H7B	108.6	C10—C20—H20A	109.5
H7A—C7—H7B	107.6	C10—C20—H20B	109.5
C14—C8—C9	119.9 (5)	H20A—C20—H20B	109.5
C14—C8—C7	118.2 (4)	C10—C20—H20C	109.5
C9—C8—C7	121.8 (5)	H20A—C20—H20C	109.5
C8—C9—C11	117.6 (5)	H20B—C20—H20C	109.5
C8—C9—C10	122.4 (4)	O1—C21—H21A	109.5
C11—C9—C10	120.0 (4)	O1—C21—H21B	109.5
C9—C10—C20	105.9 (4)	H21A—C21—H21B	109.5
C9—C10—C5	107.8 (4)	O1—C21—H21C	109.5
C20—C10—C5	114.4 (5)	H21A—C21—H21C	109.5
C9—C10—C1	111.3 (5)	H21B—C21—H21C	109.5
			
C10—C1—C2—C3	−55.4 (8)	C6—C5—C10—C1	176.2 (5)
C1—C2—C3—C4	55.2 (9)	C4—C5—C10—C1	−51.6 (7)
C2—C3—C4—C18	−168.5 (6)	C2—C1—C10—C9	170.0 (5)
C2—C3—C4—C19	76.0 (7)	C2—C1—C10—C20	−73.3 (6)
C2—C3—C4—C5	−51.4 (7)	C2—C1—C10—C5	51.8 (7)
C18—C4—C5—C6	−60.0 (6)	C8—C9—C11—C12	−0.6 (7)
C3—C4—C5—C6	−176.8 (5)	C10—C9—C11—C12	177.9 (5)
C19—C4—C5—C6	58.0 (6)	C9—C11—C12—C13	0.4 (8)
C18—C4—C5—C10	168.7 (5)	C9—C11—C12—Br	177.2 (4)
C3—C4—C5—C10	51.8 (6)	C11—C12—C13—C14	0.1 (7)
C19—C4—C5—C10	−73.3 (6)	Br—C12—C13—C14	−176.5 (4)
C10—C5—C6—C7	−65.9 (6)	C11—C12—C13—C15	177.5 (5)
C4—C5—C6—C7	160.2 (5)	Br—C12—C13—C15	0.9 (7)
C5—C6—C7—C8	40.9 (7)	C12—C13—C14—C8	−0.5 (8)
C6—C7—C8—C14	169.8 (5)	C15—C13—C14—C8	−177.9 (5)
C6—C7—C8—C9	−10.7 (8)	C9—C8—C14—C13	0.3 (8)
C14—C8—C9—C11	0.2 (7)	C7—C8—C14—C13	179.8 (5)
C7—C8—C9—C11	−179.3 (5)	C14—C13—C15—C17	86.4 (6)
C14—C8—C9—C10	−178.2 (5)	C12—C13—C15—C17	−90.9 (6)
C7—C8—C9—C10	2.3 (8)	C14—C13—C15—C16	−37.3 (7)
C8—C9—C10—C20	98.6 (5)	C12—C13—C15—C16	145.4 (5)
C11—C9—C10—C20	−79.7 (6)	C21—O1—C18—O2	−1.8 (10)
C8—C9—C10—C5	−24.1 (7)	C21—O1—C18—C4	179.7 (5)
C11—C9—C10—C5	157.5 (5)	C3—C4—C18—O2	−117.3 (7)
C8—C9—C10—C1	−142.5 (5)	C19—C4—C18—O2	1.6 (9)
C11—C9—C10—C1	39.1 (6)	C5—C4—C18—O2	125.3 (7)
C6—C5—C10—C9	55.8 (6)	C3—C4—C18—O1	61.1 (6)
C4—C5—C10—C9	−172.0 (5)	C19—C4—C18—O1	179.9 (5)
C6—C5—C10—C20	−61.7 (6)	C5—C4—C18—O1	−56.4 (6)
C4—C5—C10—C20	70.6 (6)		

### Hydrogen-bond geometry (Å, °)

**Table:**

*D*—H···*A*	*D*—H	H···*A*	*D*···*A*	*D*—H···*A*
C21—H21A···O2^i^	0.96	2.72	3.652 (10)	165

Symmetry codes: (i) *x*, *y*+1, *z*.
